# The Role of Cytokines in the Development and Functioning of the Hypothalamic–Pituitary–Gonadal Axis in Mammals in Normal and Pathological Conditions

**DOI:** 10.3390/ijms262211057

**Published:** 2025-11-15

**Authors:** Vasilina M. Ignatiuk, Viktoria S. Sharova, Liudmila A. Zakharova

**Affiliations:** Koltsov Institute of Developmental Biology, Russian Academy of Sciences, Vavilov Street, 26, 119334 Moscow, Russia; gwynnlynx@mail.ru (V.M.I.); l-a-zakharova@mail.ru (L.A.Z.)

**Keywords:** cytokines, maternal stress, hypothalamic-pituitary-gonadal axis, perinatal programming, maternal immune activation (MIA), cytokine signaling, reproductive programming

## Abstract

This review analyzes data on the effect of cytokines on the development of the hypothalamic–pituitary–gonadal (HPG) axis at all levels during pre- and postnatal ontogenesis under physiological and pathological conditions in humans and other mammals. Changes in cytokine physiological balance, associated with stress, inflammation, aging, and metabolic syndromes, affect the secretion of gonadotropin-releasing hormone, a key regulator of the HPG axis, as well as the secretion of pituitary gonadotropins, gonadal steroidogenesis, and gametogenesis in both males and females. Special attention is given to proinflammatory cytokines, the levels of which increase under the influence of infectious agents. Their impact on the development of the fetal brain and HPG axis is examined, as are the ensuing long-term consequences for HPG axis function. The study of cytokine contribution to the development and function of the HPG axis opens up broad prospects for reproductive disorder prevention during early ontogenesis.

## 1. Introduction

Cytokines are a group of regulatory polypeptides or proteins with a molecular mass of 5–50 kDa produced by various cells of the body. To date, more than 200 cytokines are known. Their active synthesis begins mainly after infection or tissue damage. They are characterized by pleiotropy, a lack of antigenic specificity, interchangeability, and the formation of a cytokine network [1]. The same cytokine can be synthesized by different cells in different tissues. Cytokine synthesis begins after pathogen-associated molecular patterns are recognized by the so-called Toll-like receptors (TLRs). There are 13 known types of TLRs, of which 10 types are present in humans (TLR1–10) and 12 in mice (TLR1–9, TLR11–13). The main ligands of TLR2 are lipoteichoic acid and cell wall peptidoglycans of Gram-positive bacteria, while the ligand of TLR4 is the lipopolysaccharide (LPS) of Gram-negative bacteria [2,3]. After binding to a TLR, the ligands trigger a molecular cascade, including a number of kinases and the key nuclear transcription factor of proinflammatory cytokine genes NF-kB [3,4]. LPS also triggers the adenylate cyclase cascade, which leads to an increase in the concentration of the cAMP second messenger, which is involved in the regulation of the inflammatory response level and the timely inhibition of the NF-kB cascade [5,6]. The result of these activation processes is the synthesis of proinflammatory cytokines that stimulate further inflammation [7,8]. The key proinflammatory cytokines include interleukin (IL) 1, IL-6, and tumor necrosis factor (TNFα), as well as chemokines, particularly IL-8 and monocyte chemoattractant protein-1 (MCP-1). Cytokines can affect all organs and tissues of the body, as well as the development of a local inflammatory response and tissue regeneration.

During embryonic development, cytokines are the key mediators linking the maternal body to the embryo. They act not only on the course of the pregnancy, but also on the developmental trajectory and health of the offspring. In particular, cytokines influence a number of cellular events—viability, proliferation, differentiation, and metabolism—potentially having long-term effects on the embryo and fetus through epigenetic programming [9]. The balance of cytokines allows the fetus to develop normally and adapt to environmental changes.

The pathological processes, e.g., inflammation, are mediated by the same molecular mechanisms which regulate physiological homeostasis and normal development. In elevated concentrations, proinflammatory cytokines can have negative effects on the developing fetal body, including the formation of the hypothalamic–pituitary–gonadal (HPG) axis, disorders of which can lead to impaired reproductive potential in the born offspring [10,11,12,13]. However, despite the emergence of information on the influence of cytokines on the developing fetal organism, data on their influence on HPG axis formation in normal and pathological conditions and on the long-term consequences in postnatal ontogenesis are sparse.

To date, data on the participation of cytokines in regulating the formation and functioning of the HPG axis, as well as in the mechanisms of development of a number of pathologies, are accumulating. However, studies on human beings are not numerous. Most of the data are obtained on animal or in vitro cell culture models. This review article presents an analysis of our own and published data on the role of cytokines and their receptors in the possible mechanisms regulating the development and functioning of the HPG axis in ontogenesis in health and disease. Primary attention was paid to pro-inflammatory cytokines (Table 1).

**Figure 1:**
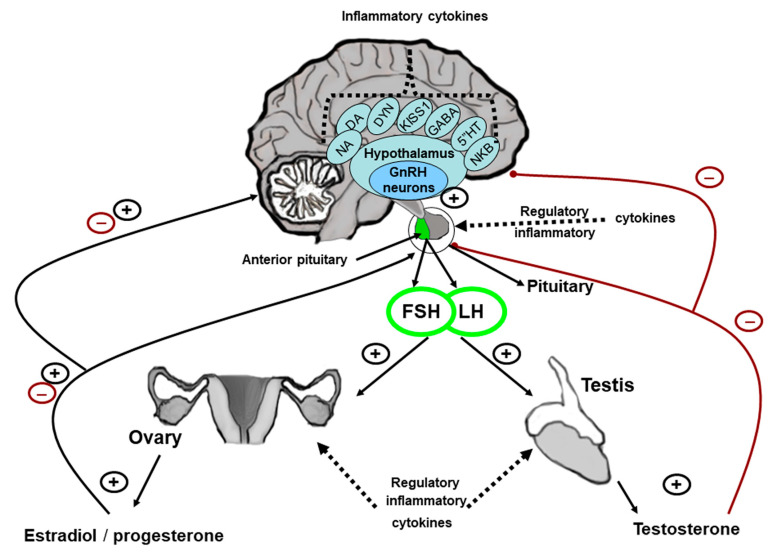
In adult mammals, the regulation of the functional activity of the hypothalamic–pituitary–gonadal (HPG) axis occurs at three levels: in the gonadotropin-releasing hormone (GnRH)-producing system in the hypothalamus, in the anterior pituitary, and in the gonads. GnRH is secreted into the portal system of the anterior pituitary and stimulates the secretion of luteinizing hormone (LH) and follicle-stimulating hormone (FSH) in the anterior pituitary. In males, gonadotropins regulate spermatogenesis and steroidogenesis. Testosterone provides feedback, inhibiting the secretion of GnRH, LH, and FSH. In females, gonadotropins regulate steroidogenesis and ovulation in the ovaries. Estradiol and progesterone provide negative and positive feedback in connection with GnRH and gonadotropins, depending on the stage of the ovulatory cycle. Different signaling molecules, serotonin (5′HT), dopamine (DA), noradrenaline (NA), gamma-aminobutyric acid (GABA), kisspeptin (KISS1), neurokinin B (NKB), and dynorphin (DYN), which are colocalized with KISS1, as well as inflammatory cytokines, are also involved in regulating the functioning of the GnRH-producing system. Regulatory and inflammatory cytokines act in the HPG axis under normal and pathological conditions. A disbalance of cytokines leads to the suppression of the HPG axis functional activity.

In sexually mature mammals, the influence of cytokines on the HPG axis is realized at all its levels: on the level of the gonadotropin-releasing hormone (GnRH)-producing system, the anterior pituitary, and the gonads (Figure 1).

## 2. The Effect of Cytokines on the Functional Activity of the GnRH-Producing System in Sexually Mature Mammals

GnRH-secreting hypothalamic neurons play a key role in the regulation of the HPG axis (Figure 1). GnRH neuron axons terminate in the median eminence, where they form axo-vasal synapses and secrete GnRH into the portal system at certain intervals. The frequency of GnRH release into the portal system determines the secretion of luteinizing (LH) and follicle-stimulating (FSH) hormones by gonadotropocytes in the anterior pituitary and is regulated by multiple neuronal systems in the brain. These include kisspeptin, the product of the *KISS1* gene, and its receptor, as well as neurokinin B, dynorphin, gamma-aminobutyric acid (GABA), and monoamines [14,15]. The frequency of tonic (in males) and cyclic (in females) GnRH secretion is regulated by the KISS1 neurons of the arcuate nucleus, which stimulate GnRH neurons directly through the synapses on their dendrons (outgrowths that have the characteristics of both dendrites and axons) [14,16]. An extensive network of afferent neurons from at least 24 different brain regions modulates the activity of KISS1 neurons. KISS1, neurokinin, and dynorphin have been shown to be involved in negative feedback formation and the maintenance of homeostasis, as well as in the regulation of the LH peak during ovulation in females [15,17].

KISS1 neurons and the associated regulatory neuronal network are central to the regulation of HPG axis activity in response to various environmental factors [16]. One such factor that significantly affects the reproductive capacity is nutrition [16,18]. Adipose tissue secretes adipokines, a group of cytokines that maintain the energy homeostasis of the body. This group includes leptin, adiponectin, and a number of other adipokines discovered in the late 1990s and early 2000s. The latter include chemerin and resistin: adipokines that are involved in the regulation of energy metabolism and also possess the properties of proinflammatory cytokines [19,20]. The role of leptin in HPG axis regulation is well understood. Leptin has the capacity to influence the HPG axis at every level, affecting the GnRH system and pituitary gonadotropocytes, stimulating GnRH receptor expression, ref. [21], as well as acting directly the gonads. Leptin suppresses testosterone secretion in the testes and thus contributes to the development of hypogonadism in overweight humans and animals [22]. Depending on its concentration, leptin can both inhibit and stimulate ovarian follicle growth [21]. At the same time, there is an inverse relationship between adipokine and sex hormone secretion: FSH and LH stimulate leptin secretion by adipocytes, while testosterone suppresses it. GnRH neurons do not express the leptin receptor. KISS1 neurons are also characterized by a low level of leptin receptor expression. The target of leptin in the HPG axis is a population of ventral premammillary neurons that form synapses on the KISS1 neurons of the arcuate nucleus and thus mediate their activity through glutamate, pituitary adenylate cyclase-activating polypeptide (PACAP), and nitrogen monoxide (NO). The maximum concentration of leptin during the ovarian cycle is synchronized with the LH peak at ovulation. Leptin also increases the expression of the receptor for GnRH on pituitary gonadotropocytes [21]. Other adipokines: chemerin, resistin, apelin and visfatin, have been shown to act on pituitary gonadotropocytes and on steroidogenesis in the gonads, and their expression in various cell populations of the central nervous system (CNS) has been detected. However, the mechanisms of their action on the GnRH system remain unexplored [20].

A number of proinflammatory cytokines, including IL-1β and IL-6, are synthesized by the glial cells of the CNS, including in the hypothalamus, and are involved in the maintenance of homeostasis. GnRH neurons express receptors to these cytokines [23]. IL-1β suppresses GnRH secretion in the medial preoptic area of the hypothalamus and hence, the ovulatory LH peak in females, whereas no such effect is observed in males [23,24]. The inhibitory effect of IL-1β in females is mediated by an increased synthesis and secretion of GABA and a decreased norepinephrine concentration in this area [25]. At the same time, there are data on the stimulation of GnRH synthesis under the action of IL-1β in vitro neuron culture. All these data confirm that cytokines can act on GnRH neurons not only directly through receptors, but also through various regulatory neuronal networks [23]. The IL-1β-induced decrease in the frequency of GnRH release may also be associated with a decrease in the activation of KISS1 neurons, but not with a decrease in the sensitivity of HPG axis neurons to KISS1 [24].

A high content of proinflammatory cytokines, characteristic of inflammatory processes, usually causes a suppression of HPG axis functions. Acute and chronic inflammatory pathologies may suppress reproductive ability. Proinflammatory cytokines can penetrate into the CNS from peripheral blood by active transport, as well as be synthesized directly in the brain by astrocytes and microglia, although the role of glial cells in modulating the expression of GnRH and KISS1 is poorly understood. During a systemic inflammatory process, the blood–brain barrier (BBB) permeability increases, including the permeability for cytokines [26]. One of the possible ways for circulating cytokines to penetrate into the CNS is via the circumventricular organs, where the BBB is absent and the neurons are in direct contact with the blood: the median eminence and the organum vasculosum of the lamina terminalis (OVLT). The somata and dendrites of GnRH neurons in the OVLT region can receive signals from the blood without disrupting BBB permeability [27]. Inflammation activation by LPS leads to a decreased expression of both GnRH and its receptor in the hypothalamus of female sheep [28,29] and rats [30].

The main cytokines involved in realizing the effect of LPS on the GnRH system are IL-1β and TNFα. In response to LPS, both cytokines exert almost similar inhibitory effects on GnRH and LH secretion [28,30,31], resulting in impaired estrous cyclicity in females [32]. Injection of IL-1β into the ventricular cavity of the rat brain significantly reduces GnRH synthesis and secretion in the septo-preoptic area in males [33]. IL-1β has been shown to suppress cfos protein expression in the nuclei of GnRH neurons, thereby altering GnRH synthesis during proestrus in rats [27]. TNFα also suppresses GnRH expression during acute and chronic indolent inflammation observed in obesity or early aging [27]. In addition, TNFα suppresses both the expression of the receptor to KISS1 in human GnRH neuron culture and LH expression in females, via KISS1 neurons and dynorphin-expressing neurons [29,34]. Data on the effects of IL-6 on GnRH neuron function in sexually mature animals are sparse and contradictory [28]. IL-6 suppresses GnRH secretion in females only slightly, and this is true both for physiological concentrations and for high concentrations [30]. However, a mediated and delayed effect of IL-6 on the HPG axis regulatory network can be hypothesized as IL-6 is involved in the regulation of synaptic plasticity, including memory and cognitive processes [35].

While proinflammatory cytokines usually cause a suppression of GnRH neuron functions, anti-inflammatory cytokines, on the contrary, stimulate the latter, maintaining physiological balance. GnRH neurons express receptors for the anti-inflammatory cytokine IL-10. IL-10 deficiency is associated with estrous cyclicity suppression and disorders of fertilization and gestation. According to Barabás et al. (2020) [27], IL-10 may be involved in the maintenance of a normal estrous cycle during an infectious-inflammatory process in the body. Moreover, transforming growth factor (TGFβ) and insulin-like growth factor (IGF-1) are noteworthy anti-inflammatory cytokines that stimulate GnRH expression [36,37]. As for interferon (IFNγ), there is insufficient evidence of its direct effects on GnRH expression. However, IFNγ stimulated monoaminergic activity in hypothalamic paraventricular nucleus, which, in turn, takes part in regulation of KISS1 and GnRH expression [38].

## 3. The Effect of Cytokines on the Functional Activity of the Anterior Pituitary

The data accumulated to date on the effect of cytokines on the functional activity of the pituitary are limited and contradictory, as they have been obtained using different experimental models, which often employ tumor lineage cells. The effects of cytokines on these cells may differ from those observed in experiments performed on healthy animals [39,40].

A number of pro- and anti-inflammatory cytokines, including IL-1, IL-2, IL-6, TNFα, TGFβ, macrophage migration inhibitory factor (MIF), and a number of others, participate in the auto- and/or paracrine regulation of the anterior pituitary function [41].

The proinflammatory cytokine IL-1, including both of its isoforms: IL-1α and IL-1β, suppresses the proliferation of most pituitary cell types [40,41]. IL-1β suppresses prolactin secretion and stimulates LH and adrenocorticotropic hormone (ACTH) secretion in rat pituitary cell culture [42]. IL-1β also induces IL-6 secretion by anterior pituitary cells, activating lysophosphatidylcholine, protein kinase C, and cAMP signaling pathways [42].

IL-1β targets are several types of pituitary cells, including follicular stellate cells (FCs). The FCs of the anterior pituitary that does not secrete hormones are connected by gap junctions and provide intercellular communication and regulation of endocrine cell activity through a number of growth factors and cytokines [43]. IL-1β regulates the expression of follistatin and inhibin/activin-β-B and suppresses FSH secretion in response to activin A. Follistatin mRNA is present in most pituitary cells, and FCs are a major source of the peptide. Activins stimulate FSH synthesis in the pituitary, while follistatin suppresses it. It has been suggested that IL-1β may modulate gonadotropic responses to activins and suppress FSH secretion by affecting the local balance of activin-B and follistatin in the anterior pituitary [44]. Moreover, activin, inhibin and follistatin may act as feedback mediators on hypothalamic level. Activin stimulates KISS-1 expression in rat brain culture, while inhibin A and follistatin suppress KISS-1 expression. However, the effects of activin, inhibin A and B subunits and follistatin may depend on the cell culture model [45,46].

One of the key cytokines secreted by FCs is IL-6, which is involved in the regulation of endocrine cell proliferation [41]. Along with the classical activators, such as IL-1, TNFα, and LPS, IL-6 secretion by FCs is stimulated by PACAP, receptors for which have been found in the pituitary and ovaries. Estrogens suppress IL-6 secretion in response to PACAP, thus participating in the formation of a feedback loop [47].

IL-6 stimulates the secretion of LH, FSH, ACTH, and somatotropin [47], and also activates the transition of anterior pituitary stem cells from dormancy to proliferation. It is notable that the effect of IL-6 is more pronounced in young animals [48]. In addition, IL-6 is involved in autocrine regulation of somatotropic pituitary cell senescence and in suppression of tumor growth [49], as well as stimulating the reparative proliferation of pituitary cells upon injury [50].

In response to corticotropin-releasing hormone (CRH), both pituitary FCs and corticotrophs secrete the regulatory cytokine MIF [51], known as an antagonist of glucocorticoid proinflammatory effect [52].

Interferon (IFNγ) suppresses corticotroph proliferation [39] and ACTH secretion [53] through the activation of JAK-STAT1 and NFkB-signaling pathways [39]. Receptors for TNFα have been found on endocrine cells of the anterior pituitary [41]. TNFα suppresses pituitary hormone secretion in response to hypothalamic releasing hormones, including GnRH [54].

In rat anterior pituitary cell culture, the pleiotropic cytokine IL-2 suppresses LH and FSH secretion, but stimulates ACTH and thyreotropin secretion [55]. IL-2 also suppresses somatotropin secretion in healthy people, which is more pronounced at a young age [56].

Cytokines such as leukemia inhibitory factor (LIF) and chemokine IL-8 (a ligand of the CXCR2 receptor) are also involved in regulating anterior pituitary function. Mutant mice with CXCR2 receptor knockout exhibit LH and FSH deficiency and reduced reproductive capacity [57]. In mutant mice with a deficiency in LIF or its receptor, ACTH secretion in response to CRH is reduced and corticotropocyte differentiation is impaired. Elevated LIF, in turn, leads to an overproduction of ACTH and glucocorticoids, as well as to a somatotropin deficiency [58].

Both acute and chronic inflammation induced by bacterial LPS or viruses is accompanied by increased levels of proinflammatory cytokines: IL-1β, IL-6, and TNFα, as well as of their receptors in the anterior pituitary [53,59]. This leads to a suppression of LH-β receptor expression and to LH secretion into the blood by gonadotropocytes, as well as to a decreased expression of the GnRH receptor in the anterior pituitary [59]. At the same time, proinflammatory cytokines, activating the HPG axis, induce adrenal gland secretion of glucocorticoids (cortisol in humans and corticosterone in rodents), which limit inflammation processes [53]. By binding to specific receptors in the brain, glucocorticoids suppress the increased secretion of so-called stress hormones in the pituitary gland (CRH, ACTH, and a number of others) and thus suppress the stress response caused by inflammation.

Blockade of TLR4, whose ligand is LPS of Gram-negative bacteria, restores LH and FSH secretion suppressed during inflammation [60]. Acetylcholine esterase inhibitors, which activate the anti-inflammatory cholinergic pathway, also restore LH and FSH secretion [61].

## 4. The Effect of Cytokines on the Functional Activity of the Gonads in Sexually Mature Males

It is known that cells of innate immunity, and primarily macrophages, take an active part in the normal regulation of spermatogenesis and Leydig cell steroidogenic function [62,63]. The question of the mechanisms of interaction between these cells has been open for a long time, and interest in it has been renewed only in recent years. Among testicular macrophages, two subpopulations are distinguished: interstitial and peritubular [64]. Interstitial testicular macrophages account for about 20% of the interstitial testicular tissue compartment [65]. They form cytoplasmic interdigitations with Leydig cells, characteristic only for sexually mature mammals [66]. If there is a testicular macrophage deficiency, caused by selective local deletion or knockout of the colony-stimulating factor 1 (*CSF1*) gene, which is required for macrophage differentiation, suppression of Leydig cell differentiation in the juvenile period is observed [66,67]. In sexually mature rodents, macrophages are essential both for maintaining normal testosterone secretion in response to gonadotropins [65,67] and for regulating spermatogenesis [65]. Macrophages are involved in the transport of testosterone precursors to Leydig cells and perform their paracrine regulation [68]. In turn, testosterone reduces the production of proinflammatory cytokines by testicular macrophages, thus maintaining a negative feedback loop [66]. Leydig cells secrete CSF1, a cytokine that not only plays a key role in the regulation of macrophage differentiation, but also participates in the maintenance of spermatogonia proliferation. Testicular macrophages do not express CSF1 but can regulate its concentration by receptor-mediated endocytosis [66]. They also express enzymes of retinoic acid synthesis, which stimulates spermatogonia differentiation [65].

The interaction between macrophages and Leydig cells can be realized through pro- and anti-inflammatory cytokines [63,68]. The effect of the latter depends on a number of factors: concentration, exposure time, and age. In the male gonads, IL-6 and IL-1 are synthesized by macrophages, as well as by Sertoli and Leydig cells [69]. In normal conditions, the balance of these cytokines is regulated by the anti-inflammatory cytokines IL-10 and TGFβ [70]. For example, IL-1β at high doses (10 ng/mL) suppresses the testosterone secretion and viability of murine Leydig cells when the cells are cultured for 48 h [71]. At the same time, at low doses, IL-1β (1–2 ng/mL) stimulates the proliferation of Leydig cells obtained from rats in the prepubertal period, whereas no such effect has been observed in the pubertal period [66]. The effect of IL-1β on the steroidogenic function of Leydig cells is also more pronounced in the prepubertal period than in the pubertal period [72,73]. The IL-1α isoform stimulates the steroidogenic activity of Leydig cells much more strongly than IL-1β [72].

Leydig cells, in turn, synthesize the cytokine MIF [68,74]. MIF binds to the CD74 receptor of macrophages and activates the NLRP3 (Nod-like receptor protein 3) protein complex with caspase 1, which cleaves pro-IL-1β to active IL-1β [75].

Specific inhibition of IL-1β and TNFα leads to the suppression of the spermatogenic as well as steroidogenic functions of the testes in rodents [68,76]. TNFα, synthesized by testicular macrophages and spermatids [77], stimulates androgen receptor expression in Sertoli cells and suppresses anti-Müllerian hormone synthesis in them [78,79]. In addition, TNFα regulates the viability of developing germ cells by blocking Fas-mediated apoptosis [77,80]. During chronic inflammation, TNFα, IL-1β, and IL-6 dose-dependently suppress the steroidogenic function of Leydig cells, with IL-6 also regulating their viability [71,81]. At the same time, IL-8 enhances Leydig cell viability and growth in vitro culture. Elevated levels of IL-6 have been reported in infertile men [82].

IL-6 is synthesized in response to FSH and testosterone by Sertoli cells [83,84], in which it regulates the rearrangement of tight junction proteins and, consequently, the permeability of the blood–testis barrier. This process has been shown to be mediated by the mitogen-activated protein (MAP) kinase pathway [85].

Peritubular testicular macrophages participate in the maintenance of the blood–testis barrier and the immunoprivileged status of the testes. They induce the differentiation of naive T lymphocytes into T regulatory cells to form systemic tolerance to germ cell antigens [86,87]. They are characterized by a low secretion of proinflammatory cytokines and secretion of anti-inflammatory cytokines such as TGFβ and IL-10 [64,87].

During inflammation, IL-1β and TGFβ are mostly presented in the testes by their inactive forms, and enzymatic processing appears to be an important control mechanism for these two cytokines [88]. The authors suggest that in the testes, regulation of the activity of both proinflammatory and regulatory cytokines occurs not only at the level of gene expression, but also at the level of post-translational modification.

Along with interleukins, Sertoli cells and peritubular cells synthesize activin, a cytokine from the TGFβ family, which has both pro- and anti-inflammatory effects, as well as its antagonist inhibin. Activin regulates the response of Sertoli cells to FSH. When stimulated by proinflammatory agents such as bacterial LPS or IL-1, activin expression is upregulated. The balance of activin and inhibin expression by Sertoli cells depends on the stage of spermatogenic epithelium: the maximum level of activin expression is observed at the spermiation stage [89]. It is thought that the activin and inhibin balance plays a role in regulating the viability, proliferation, and meiosis of spermatogenic cells [90]. The stage of spermiation, when Sertoli cells phagocytize spermatid residual cytoplasm, is also characterized by a maximum level of expression of IL-1α and IL-6 by these cells [89]. Prolonged exposure to activin A causes macrophages to switch to an anti-inflammatory phenotype and triggers regulatory T lymphocyte differentiation [91]. This suggests that activin A is also involved in the regulation of the immunoprivileged status of the testes.

During acute inflammatory processes, proinflammatory cytokines in high concentrations suppress testosterone secretion in response to gonadotropins [92]. Testosterone concentrations are reduced not only in the circulating blood, but also in the seminal plasma of the testes, which plays an important role in maintaining spermatogenesis [64,93]. The auto- and paracrine regulation of testicular macrophages may be impaired. A chronic inflammatory process caused by bacterial or viral infections of the urogenital tract is considered as one of the possible causes of male infertility [94].

The immune response to pathogens is realized through TLRs, the expression of which has been detected on macrophages. Peritubular myoid cells involved in the regulation of spermatogenesis also express TLRs [95] and are capable of synthesizing pro- and anti-inflammatory cytokines [96].

Testicular macrophages are characterized by reduced TLR expression and impaired ubiquitination and degradation of IĸBα-kinase, an inhibitor of the transcription factor NF-ĸB, all of which ultimately inactivates the inflammatory signaling pathway [87].

Blood supply disorders of the testes, including varicocele and testicular torsion, cause aseptic inflammation and may also be responsible for decreased fertility [97]. Damaged or destroyed spermatogenic cells can also activate the immune response by stimulating the expression of IL-1β, IL-6, TNFα, and MCP-1 [98]. Elevated concentrations of proinflammatory cytokines such as IL-1β and TNFα in the seminal fluid correlate with impaired spermatogenesis and infertility [99]. Extracellular ATP (adenosine 5′-triphosphate), released by damaged cells and by the activated cells of the immune system, stimulates the synthesis of proinflammatory cytokines by peritubular myoid cells [96]. At the same time, the integrity of the blood–testis barrier is compromised [85,87]. A significant number of monocytes from the blood are attracted to the focus (of inflammation, and the cytokine expression profile of testicular macrophages is also altered [87]. The oxidative stress that accompanies acute and chronic inflammatory processes is also a factor that damages both spermatogenic cells [100] and Sertoli and Leydig cells [101]. Increased macrophage secretion of cytokines, such as activin A, TGFβ, and chemokine CCL2, as well as matrix metalloproteinases, stimulates fibroblast proliferation and collagen and fibronectin synthesis, ultimately leading to testicular fibrosis [89,102].

## 5. The Effect of Cytokines on the Functional Activity of the Ovaries in Sexually Mature Females

Pro- and anti-inflammatory cytokines are involved in the regulation of ovarian cyclicity in mammals at all stages, including follicular growth, ovulation, luteinization, follicular atresia, and luteolysis. Thus, TNFα and IL-1β suppress steroid secretion in response to gonadotropins in undifferentiated ovarian cells through the inhibition of the cAMP pathway [103]. TNFα inhibits aromatase expression in ovarian granulosa cells and reduces their viability and proliferative activity in culture [104]. In addition, TNFα and IFNγ inhibit the local synthesis of connective tissue growth factor and thus induce the apoptosis of granulosa cells. IL-1α and IL-1β reduce gonadotropin receptor expression on granulosa cells [105]. In the process of corpus luteum formation, they stimulate lutein cell proliferation and inhibit progesterone secretion. At the same time, IL-1β stimulates progesterone synthesis in a culture of differentiated human granulosa-lutein cells [103]. In response to IL-1β, the increase in the expression of steroidogenic acute regulatory protein (StAR), which is involved in progesterone biosynthesis, is comparable to the effect of LH. The effect of IL-1β is mediated by the p38 and cAMP response element-binding protein cascade [106]. The local source of IL-1β in the ovary can be immune cells or granulosa cells.

The active form of IL-33 from the IL-1 family, which is formed during tissue damage and inflammation, is also released during ovulation and follicular atresia. However, its blockade by antibodies does not suppress these processes [107]. IL-33 is thought to be involved in the regulation of autophagy and is required for the removal of degenerating tissues during atresia. Mutants with IL-33 gene knockout accumulate senescent tissues, and the structural integrity of the ovary is disrupted as a result [108].

The ovary, like the testis, is characterized by a population of resident tissue macrophages [109]. When the macrophages are removed, steroidogenesis is suppressed, and the integrity of ovarian blood vessels is compromised [109]. Under the influence of the microenvironment, macrophages, including ovarian macrophages, can differentiate into two types that differ in their cytokine expression profile. Type M1 is characterized by classical activation and the synthesis of proinflammatory cytokines, as well as the elimination of pathogens and destroyed cells. Type M2 is involved in intercellular matrix remodeling and tissue repair [110]. During ovulation, M1 macrophage differentiation and an increased synthesis of cytokines characteristic of this type (IL-1β and IL-6) predominate. After 48 h, when the corpus luteum is formed, the expression of these cytokines decreases to the control level. M2 macrophages expressing TGFβ surround the growing follicles, and M1 macrophages participate in the activation of primordial follicles. Along with the cytokines synthesized by macrophages, follicle development is also regulated by extracellular vesicles containing specific microRNAs [111].

It has long been known that the process of ovulation has similarities with an acute inflammatory response: the former includes the synthesis of proinflammatory cytokines, infiltration by lymphocytes and macrophages, the formation of reactive oxygen species, and prostaglandin synthesis [111]. Ovulation can be suppressed by anti-inflammatory drugs as well as antioxidants [112]. At the same time, unlike pathological inflammation, the physiological process of ovulation is tightly controlled and is suppressed in a timely manner by a negative feedback system to avoid tissue damage and the development of autoimmune processes [107].

At the ovulation stage, IL-6 [113] and TNFα expression is significantly increased in cumulus cells that form the oocyte microenvironment during follicle maturation [114]. IL-6, bound to the soluble form of the receptor (sIL-6Rα), activates the JAK2/STAT3 cascade and the expression of prostaglandin endoperoxide synthase, a key enzyme for the synthesis of the inflammatory mediator prostaglandin E2 [113]. Prostaglandin E2 stimulates the expression of proteases required for the separation of the cumulus from the follicle wall and the rupture of the follicle wall, and is involved in the regulation of oocyte maturation [115]. Prostaglandin E2 is also an antagonist of chemokines, particularly CCL7, which stimulate the synthesis of the extracellular matrix by the cumulus cells, thus ensuring the permeability of the cumulus to spermatozoa [116]. The number of immune cells in the cumulus is very low compared with the number of cumulus cells, which are the main producer of chemokines [117].

The follicular fluid contains the anti-inflammatory cytokine TGFβ, receptors for which are expressed on granulosa cells. TGFβ prevents premature luteinization and follicular atresia. It reduces StAR expression and the biosynthesis of progesterone by granulosa cells in the follicular phase, a premature increase in which in the follicular fluid causes follicular atresia [118]. TGFβ also suppresses the expression of genes related to luteinization, in particular matrix metalloproteinases [119]. In granulosa cell culture, TGFβ increases the expression of connexin (gap junction protein). Thus, TGFβ plays an important role in intercellular communication and the synchronization of follicular cell activity [120]. In addition, TGFβ suppresses microvasculature-like formation and the expression of vascular-endothelial-cadherin (the adhesion protein of endothelial cells) in human granulosa-lutein cell culture [121]. Amphiregulin, which is an epidermal growth factor receptor ligand [122], is an antagonist of TGFβ and stimulates vascularization during corpus luteum formation. Amphiregulin expression in the follicle wall dramatically increases in response to LH [123]. The balance of these two factors ensures the suppression of granulosa vascularization (in the follicular phase, vessels are present only in thecal layer) and the vascularization of the corpus luteum [113,121].

In response to LH, the expression of the chemokine CCR2 receptor, whose key ligand is MCP-1, is upregulated in cumulus cells. MCP-1 and the stromal cell-derived factor (SDF1) chemokine [123] upregulate the expression of ovulatory cascade genes, including amphiregulin. Moreover, the effect of MCP-1 is more pronounced at high concentrations than at low concentrations [123].

KISS1, which is the key regulator of GnRH pulse release, also regulates the activation of primordial follicles in the ovary [124] and is involved in regulating the ovarian response to pituitary gonadotropins. KISS1 deficiency causes the suppression of antral follicle growth, ovulation, and luteinization, even in cases where gonadotropins are administered [125]. The content of KISS1 in rat ovaries changes throughout the ovarian cycle, reaching a maximum at the proestrus stage, immediately preceding ovulation. A decrease in LH levels is accompanied by a decrease in KISS1 content; while after chorionic gonadotropin administration, its increase is observed [126]. Ovarian tissues, including theca, granulosa, corpus luteum, and oocyte cells, express KISS1 receptor [124,127]. However, the available data on KISS1 receptor expression in different ovarian tissues are inconsistent and depend on the age of the animals and the stage of the ovarian cycle. In humans, the expression of this receptor increases with follicle growth and reaches its maximum by the ovulation stage [124].

A shift in the physiological balance between pro- and anti-inflammatory cytokines towards proinflammatory cytokines characterizes both normal and pathological aging of the gonads [128]. The expression of proinflammatory cytokines, including IL-1β, TNFα, IL-6, IL-8, and IL-18, increases with ovarian aging and is a marker of a premature loss of fertility [128,129]. At the same time, there is a decrease in the mRNA content of the anti-inflammatory cytokine IL-10, an increase in the synthesis of cyclooxygenase, and, consequently, an increase in the content of reactive oxygen species. Estrogens can exert systemic anti-inflammatory effects by increasing the synthesis of and inhibiting the degradation of the IkB kinase complex. Reduction in estrogen levels leads to pro-inflammatory cytokine elevation and visceral fat accumulation, associated with normal and pathological aging [130].

Polycystic ovary syndrome (PCOS) is the most common ovarian pathology and a frequent cause of infertility. This syndrome is associated with a disorder of the HPG axis feedback system. PCOS is characterized by elevated circulating androgen levels, an impaired carbohydrate and lipid metabolism (i.e., insulin resistance and obesity), and a lack of ovulation [131]. PCOS is often characterized by rapid pulsations in the release of GnRH, an elevated LH/FSH ratio [132], and increased blood levels of KISS1 [131]. Elevated leptin and inhibin levels are also considered as potential markers of PCOS [131]. PCOS is distinguished by chronic mild inflammation and oxidative stress. The content of proinflammatory cytokines, including IL-18, MCP-1, TNFα [133], and IL-1 [134], increases, as does the activity of the transcription factor NF-kB [133]. Moreover, NF-kB pathway suppression ameliorates PCOS prominence [135].

Hyperglycemia is a factor that activates NF-kB, which consequently leads to the expression of proinflammatory cytokines, including TNFα and IL-6 [136]. TNFα stimulates the proliferation of theca cells [137] and their steroidogenic activity [138], as well as decreasing tissue sensitivity to insulin, enhancing the development of insulin resistance [136]. TNFα suppresses estradiol secretion, while elevated androgen levels, in turn, increase the secretion of TNFα and other proinflammatory cytokines, forming a “vicious circle” [138,139]. Concurrently, specific inhibition of TNFα reduces the expression of the PCOS phenotype and normalizes follicle differentiation [140].

Under normal conditions, the capacity of macrophages to become activated changes throughout the ovulatory cycle. This capacity peaks at ovulation, when macrophages infiltrate ovarian tissue. The cyclicity of macrophage activation capacity is less prominent in the case of PCOS [139]. The spleen macrophages of PCOS mice stimulate androgen secretion and suppress estradiol secretion in granulosa cell culture [141]. Chemerin, a cytokine from the group of adipokines secreted by adipocytes, is involved in the regulation of macrophage activation. Chemerin stimulates the differentiation of M1 macrophages and suppresses that of M2 macrophages [142]. PCOS is characterized by an increase in circulating chemerin concentration, which is associated with insulin resistance and excess adipose tissue [143]. Adipose tissue is also a source of IL-6 and TNFα [142]. Obesity concomitant with PCOS increases the severity of the symptoms. IL-15, a pro-inflammatory cytokine involved in the chronic low-grade inflammatory process associated with obesity, suppresses granulosa cell survival and steroidogenic activity [144]. Increased concentrations of the cytokine MIF have been observed in the plasma [145] and in ovarian tissue [146] during PCOS. IL-10 secreted by mesenchymal stem cells, on the contrary, decreases the intensity of the inflammatory process and reduces androgen synthesis, thus leading to increased fertility during PCOS [147].

Thus, sufficient evidence has been obtained regarding the role of various cytokines in regulating the function of the main elements of the HPG axis under normal and pathological conditions. At the same time, studies on the influence of cytokines on development, including the HPG axis in fetuses, are still in the early stages of experimentation, data collection, and analysis.

## 6. The Effect of Cytokines on the Developing Fetus

Cytokines significantly impact the course of maternal gestation and the development of the fetus, thereby determining the offspring’s phenotype and health [9]. In recent decades, researchers have paid particular attention to the role of cytokines in the development of various brain structures in the offspring [10,148]. Cytokines have been shown to be involved in all stages of brain development, functioning as morphogenetic and regulatory factors. Cytokines are involved in the regulation of gliogenesis, synaptic transmission, neural plasticity, and neuroepithelium proliferation [13,149]. Cytokine receptors are found in different parts of the brain, including the cortex, the hippocampus, and the hypothalamus in normal development [150]. Therefore, the alterations of cytokine levels, associated with pathological conditions, may disorder the fetal development.

In response to inflammation, microglial cells begin to synthesize large amounts of cytokines [148,151]. Cytokines are synthesized by astrocytes and neurons, along with microglial cells. The so-called “olfactory ensheathing cells” (OECs), which form a migratory mass with other types of neurons, contribute to the regulation of GnRH neuron development [152,153]. This type of glia contains a wide range of cytokine receptors [154]. Cytokines induce metabolic reprogramming of glial cells and influence olfactory neuron development [155]. Inflammatory processes cause an impairment of GnRH neuron intranasal migration [12], which can be explained by a modulation of OEC phenotypes by cytokines. OECs, in turn, can influence activated microglia [151], reducing the detrimental effects of neuroinflammation on the development of GnRH neurons [156].

Increased expression of pro- and anti-inflammatory cytokines in both mothers and fetuses, induced by LPS, leads to developmental disorders and preterm labor [12,13,156,157]. Changes in fetal brain development increase the risk of various psycho-neurological disorders during postnatal ontogenesis. Important factors in fetal brain development are the period of fetal and placental development and the dosage of the agent activating the maternal immune response. Thus, in mice, a high immune response can be mediated either by a low dose of LPS or by administering it during the early stages of pregnancy, from embryonic day (ED) 0 to embryonic day 11.5. Conversely, a low immune response may be associated with a higher dose of the agent or with its administration later in the pregnancy [158]. A delayed intranasal migration of GnRH neurons after LPS administration to female rodents on ED12 results in an impaired formation of afferent innervation in adult animals [13,157]. A decrease in the number of synapses that regulate GnRH neuron pulsatile activity [14] causes disturbances in the formation of the HPG axis feedback network. As a result, females experience delayed puberty, decreased circulating LH and estradiol levels, as well as increased ovarian follicle atresia [157], while males experience decreased circulating FSH and testosterone levels and may be subject to spermatogenesis failure [13,159]. Such disorders are not observed after exposure to LPS on ED18, i.e., later in the pregnancy [13]. Suppressing the inflammatory process with polyclonal immunoglobulin (IgG) 40 min after LPS exposure, when the secretion of proinflammatory cytokines has not yet reached its peak, reduces the severity of the observed disorders [13,157,160]. Although the mechanism of the anti-inflammatory action of IgG is currently poorly understood, IgG is widely used to treat infectious and autoimmune diseases, as well as recurrent miscarriage [161].

Following LPS exposure on ED11, a decrease in the number of dopaminergic neurons in the substantia nigra and an increase in microglia activity and proinflammatory cytokine synthesis, most notably TNFα, were observed in sexually mature rat offspring. It has been suggested that LPS-induced neuroinflammation leads to the suppression of the secretion of glutathione, which exhibits anti-oxidant properties, in glial cells. This process can cause the death of dopaminergic neurons and subsequent development of Parkinson’s disease [162].

One hour after prenatal LPS exposure, the expression of TNFα and IL-1β was observed in the rat fetal brain. At the same time, the level of TNFα was maintained over the next 24 h, whereas the content of IL-1β decreased [163]. The maximum expression of these cytokines is noted in the first days of postnatal development.

Under normal physiological conditions, IL-1β stimulates the secretion of the neurotrophic factor neurotrophin-3 (NT-3) and the transcription factor neurogenin-1 (Ngn1) involved in cranial nerve development and neuronal network expansion. IL-1β is also involved in regulating the synthesis of specific proteins, in particular Wnt5a, which in turn prevents cell differentiation through the formation of small interfering RNAs (siRNAs) [164]. In vivo, IL-1β suppresses GnRH secretion, but in vitro, it stimulates it [23].

According to Rousset et al., significant IL-1β expression is observed 3 days after LPS administration on postnatal day 1 (PND1). By PND7, astrogliosis and significant hypomyelination in the CNS are observed [165]. During inflammatory processes in rats, TNFα and IL-1β primarily disorder the development of fetal brain white matter. This may later lead to the development of cerebral palsy and schizophrenia diagnostic signs [166]. However, it should be noted that, 30 min after the administration of IL-1β to the mother, only 1% of the labeled IL-1β concentration in the placenta reaches the fetal brain and only 3% reaches the fetal liver [167].

The proinflammatory cytokine IL-18, which induces IFNγ synthesis, belongs to the IL-1 family. Expression of the IL-18 receptor has been detected on GnRH neurons in the mouse and rat brain [168,169]. Neuronal somata and fibers containing IL-18 and its receptor (IL-18Rα) are found throughout the medial septal nucleus, the preoptic area, and the anterior hypothalamus, where GnRH neuron somata and fibers are mainly located. Approximately 60% of GnRH somata contain IL-18Rα, and all GnRH somata contain IL-18.

**Figure 2:**
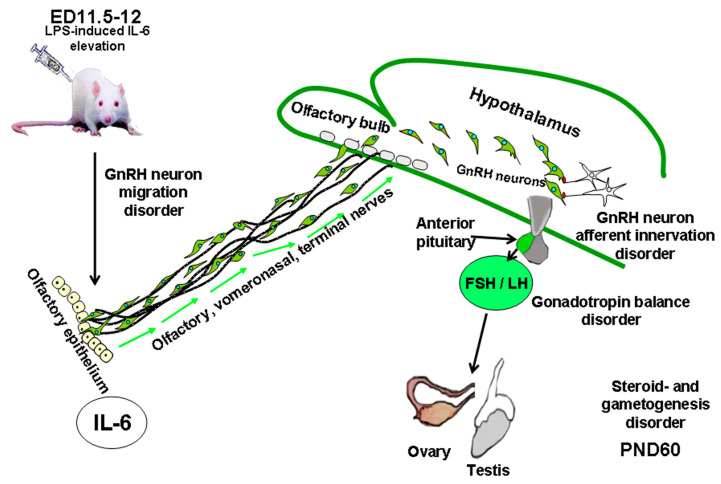
Systemic inflammation induced by IL-6 on embryonic days (ED) 11.5–12 causes disturbances in the development of the GnRH-producing system in rodents. Elevated levels of IL-6 slow down the intranasal migration of GnRH neurons, eventually disrupt the formation of their synaptic inputs in the hypothalamus, and change the pulse frequency of GnRH releases into the portal system of the anterior pituitary. Gonadotropins, specifically luteinizing hormone (LH) and follicle-stimulating hormone (FSH), regulate gonadal functions. Gonadotropin disbalance results in impaired spermatogenesis, estrous cycle, and sexual behavior on postnatal day (PND) 60.

IL-6 has a significant effect on brain development. In physiological concentrations, it regulates neuronal proliferation and differentiation, axonal growth [12,170], and synaptic pruning [171], but its elevated levels lead to cell death [170]. IL-6 is involved in the regulation of neuro- and gliogenesis, acting directly on progenitor cells [172]. Recent studies have shown that maternal IL-6 levels correlate with the formation of synaptic inputs in the brain and memory development of the child over a 2-year period starting from the neonatal period [173]. IL-6 is thought to be a major potential mediator linking maternal immune system activation during pregnancy and further development of the nervous system in the offspring [174,175,176]. Prenatal IL-6 exposure causes an increase in the number of synaptic inputs in the hippocampus and launches genetic programs of synaptogenesis in the developing neurons [171]. In mice, exposure to IL-6 on ED13.5 leads to an increase in the number of neuronal progenitors in the forebrain and to fiber growth disorders in the olfactory neuronal sub-population expressing the calcium-binding protein calretinin. These changes are eventually fixed in the offspring of mice in the long term [177]. When administered systemically, recombinant IL-6 can cause effects similar to LPS or double-stranded RNA (poly I: C) [178]. IL-6 administration to pregnant females at a dose corresponding to the IL-6 content in the blood of fetuses following LPS exposure results in a delay of GnRH neuron intranasal migration into the fetal brain (Figure 2).

Subsequently, this causes an impaired formation of synaptic connections between GnRH neurons in the hypothalamus [13,157], as well as changing the GnRH pulse frequency into the portal system of the anterior pituitary. The effect of IL-6 is realized through a specific receptor localized on the axons that form the migratory pathway of GnRH neurons. Elevated IL-6 level suppresses the growth of olfactory neural fibers and thus participates in the formation of the migration pathway of GnRH neurons [12]. Disturbances in the migration of GnRH neurons induced by inflammation during the initial stages of their exit from the olfactory placode epithelium suppress the functional activity of the HPG axis in sexually mature offspring. At the same time, restoration of GnRH neuron migration following IL-6 receptor blockade normalizes the development of the entire HPG axis: the structure and function of the ovaries and the testes and gonadotropin secretion by the anterior pituitary [13,157].

The question of whether LPS or the cytokines it induces can reach the fetus through the maternal placenta remains open, since the available data are contradictory [156,160]. Studies on animal models have shown that a number of cytokines, including IL-6, can penetrate the placental barrier and directly participate in the modulation of fetal development [179,180].

The probability of transplacental transmission increases with systemic inflammation and placental pathology. The placenta is very sensitive to proinflammatory signals, especially in the early stages of pregnancy. Even a slight activation of innate immunity in mice with low doses of LPS during this critical period causes transplacental hemorrhage and results in an elevated risk of miscarriage. The surviving fetuses show cerebral hypoxia and impaired neurogenesis. The authors associate the placental pathology with the proinflammatory cytokine TNFα, the negative effect of which is reversed by its antagonists [181]. It is noted that placental sensitivity in rodents increases from ED7 to ED11.5. Moderate LPS dose on ED12.5 causes placental damage, further fetal hypoxia, and decreased neuronal progenitor proliferation, but not fetal mortality [182]. However, exposure to high doses of double-stranded RNA (Poly(I:C)) increases fetal mortality, even in the early stages of development (ED9) [183]. By ED14.5, the mouse placenta undergoes developmental changes and becomes resistant to the effects of cytokines.

During maternal inflammatory processes, an increase in the content of IL-6 and its mRNA in the placenta is observed [178,184]. Both during a normal course of pregnancy and during inflammation, the placenta itself is a source of cytokines, particularly IL-1β and IL-6 [185,186]. Elevated IL-6 levels in the placenta may disrupt the immunological balance in the placenta by altering the ratio of Th1/Th2 subpopulations [187,188]. It is assumed that the permeability of the rat placental barrier to IL-6 is much higher in mid-gestation than in late pregnancy [180]. In addition, IL-6 can induce changes in the parameters affecting fetal growth, including nutrient transport, vascular permeability, and tissue oxygenation [189,190].

IL-6 can induce the synthesis of cytokines that also affect brain development [178]. Among them, IL-17, which is synthesized by a subpopulation of Th17 lymphocytes, is particularly noteworthy. IL-17, in turn, stimulates the synthesis of IL-6, IL-1β, TNFα, granulocyte-macrophage colony-stimulating factor (GM-CSF), granulocyte colony-stimulating factor (G-CSF), and chemokines (CCL20 and CXCL1). IL-17 activates glial cells, which begin to synthesize various proinflammatory cytokines that increase the susceptibility of the brain to destruction by cytotoxic immune cells [174]. Neutralization of elevated levels of such cytokines as IL-6 or IL-17 in pregnant females suppresses autism-like disorders in the rodent offspring [174,191].

The pleiotropic cytokine LIF is also a member of the IL-6 superfamily. It is essential for germ cell proliferation, spermatocyte differentiation, blastocyst implantation, and pituitary and olfactory system development [192]. LIF was first identified in the pituitary of cattle, and its receptors have been found in both fetal and adult pituitary [193]. In the pituitary, LIF stimulates pro-opiomelanocortin (POMC) transcription and ACTH secretion. LIF deficiency leads to suppression of ACTH secretion and to stress response [194]. At the same time, an elevated expression of LIF in transgenic mice revealed corticotroph hyperplasia and significant somatotroph and gonadotroph hypoplasia in the developing pituitary, along with multiple Rathke pouch cysts lined by ciliated cells. LIF overexpression in the Rathke pouch on ED10 redirects the differentiation of hormone-secreting cells towards the corticotroph lineage and ciliated nasopharyngeal-like epithelium [195].

LIF expression in placental cells is necessary for blastocyst implantation in mice [196]. On the model of immature and migratory GnRH neurons (line GN11), it was shown that LIF induces their chemokinesis [197]. The stimulating effect of LIF on GnRH secretion by neurons was shown in an in vitro culture [198]. LIF and its receptor (LIFβ) are expressed in the nasal area in ED13 mice, which corresponds to the stage of migration of GnRH neurons in the nasal mesenchyme. In addition, receptors to IL-6 and LIF were detected on the germinal cells of human fetal ovaries, including oocytes in primordial follicles. The expression of these receptors significantly increases with increasing gestational age [199]. The receptor to LIF was also detected on urogenital ridge cells [200].

After exogenous administration of LIF, the increase in LIF content in the blood of pregnant female rats is accompanied by an increase in the content of this cytokine in the blood and cerebrospinal fluid of fetuses [201]. These data indicate LIF transplacental transmission.

The chemokine MCP-1 is involved in the regulation of neuronal development by directing neuronal migration. In addition, MCP-1 enhances neuronal excitability and controls the synaptic function of neurons. The receptor to MCP-1 (CCR2) has been identified on GnRH neurons in the hypothalamus and most of all in the hippocampus, as well as in the GT1-7 and GN11 cell lines. In a culture of these cells, MCP-1 stimulates the migration of immature GnRH neurons [202]. The increase in MCP-1 content induced by inflammation in the mother is accompanied by an increase in MCP-1 content in the fetal brain [203], which may disorder neuronal development.

Along with MCP-1, hepatocyte growth factor (HGF) is also a chemoattractant. It promotes cell motility in the developing brain and acts as a motogen and chemoattractant on immortalized GnRH neurons (GN11). HGF and its receptor (Met) are involved in the development of the GnRH system. In rodents, HGF is detected in the nasal mesenchyme from ED12 onwards, where HGF is involved in the migration processes of GnRH neurons [204]. Its concentration increases from the nasal region to the brain [205]. During GnRH neuron migration, the neurons express the Met receptor, and its expression is suppressed after this process is completed [206]. A disruption of the HGF concentration gradient slows down GnRH neuron migration and axonal cone growth. More, knockout of the HGF activator (tPA) in mice, which is also found in GnRH neurons, leads to a decrease in the total number of these neurons [206]. In nasal explants, neutralizing HGF by specific antibodies suppresses the migration of GnRH neurons and triggers the growth of olfactory axons, which corresponds to a disruption of the concentration gradient.

In addition to their direct effect on GnRH expression and secretion, proinflammatory cytokines reduce the rate of neuronal response to kisspeptin (Kiss1), a key regulator of GnRH secretion. The axons of KISS1 neurons form a pericapillary plexus at the site of GnRH secretion [207]. Blocking the kisspeptin receptor (GPR54) in a culture of human fetal GnRH neurons suppresses TNFα expression [34].

In addition to proinflammatory cytokines, anti-inflammatory cytokines play an important role in brain development. Generally, the activity of anti-inflammatory cytokines is suppressed by proinflammatory cytokines when the immune system is activated. Overexpression of IL-10 leads to impaired behavioral responses [27]. IL-10 can affect GnRH neurons directly through a specific receptor and alter their function [27]. It has been shown that during bacterial or viral infection, IL-10 induces ERK1/2 (extracellular signal-regulated kinases 1 and 2) phosphorylation and maintains the integrity of estrous cyclicity [168]. Notably, female mice with IL-10 knockout lack ERK1/2 phosphorylation in GnRH neurons and demonstrate disrupted estrous cycles [208].

Adipokines are also involved in the regulation of development. Among them, leptin is the most studied, while data for chemerin and visfatin are sparse and mainly concern the early stages of ontogenesis: pre-implantation development and placentation [209]. During embryonic development, leptin can be transported through the placenta from the mother’s body, as well as synthesized by differentiating fetal adipocytes [210,211]. Leptin is involved in the regulation of neurogenesis and CNS development. It stimulates neuronal proliferation and neural fiber elongation [210]. The fetuses of mice with a knockout of leptin or its receptor have a reduced neural fiber density in the hypothalamus. The effect of leptin antagonists on rat fetuses varies by sex: male fetuses have an increased body weight, while the weight of female fetuses remains unchanged, yet their balance of neuronal proliferation and apoptosis is altered [210,212]. In the early postnatal period, leptin enters the body with maternal milk [210] and also regulates the formation of neuronal connections in the hypothalamus [213].

Adipokines, such as adiponectin, are synthesized by fetal white adipose tissue and maternal endometrium and are involved in regulating cytokine balance, vascularization, and the immune functions of the placenta [211]. At the same time, data on the role of adiponectin in the development of the fetal nervous system are sparse.

Exposure to stress factors, including inflammatory processes, during early ontogenesis can lead to an increased body weight in male mice and rats [214,215]. In mice, this is associated with a delayed puberty and an impaired sexual behavior [160], whereas in rats exposed to stress late in embryonic development, an increased body weight is not associated with an impaired puberty [13]. There is growing evidence that exposure to stress during early development can lead to an imbalance of adipokines, eventually leading to dyslipidemia and insulin resistance [214,216]. At the same time, a prenatal stress-induced imbalance of adipokines—increased leptin and decreased adiponectin—leads to insulin resistance without a significant increase in body weight [217].

It should be noted that the effect of leptin and other adipokines on the developing organism mainly concerns lipid and carbohydrate metabolism and eating behavior. The data on adipokine influence on the development of the HPG axis in the perinatal period are sporadic. It has been shown that body weight and lipid metabolism influence the timely onset of puberty and, as a consequence, the reproductive ability [218].

Analysis of the research literature data accumulated to date has shown that the effects of various stressor stimuli, including inflammation induced by an activation of the immune system at different stages of ontogenesis, can have a negative impact on the formation of the HPG axis.

## 7. Conclusions

The analysis of published and original data demonstrates that cytokines form an extensive class of signaling molecules that regulate the normal functioning of all components of the HPG axis and mediate its interaction with other physiological systems in adults. During prenatal ontogenesis, cytokines program the development of fetal organs and tissues; in particular, cytokines take part in regulating the intranasal migration of GnRH neuron progenitors into the brain, as well as pituitary and gonadal histogenesis and the establishment of the HPG axis feedback system. During critical developmental periods, changes in the molecular mechanisms that control the development of the HPG axis, induced by inflammation and elevated proinflammatory cytokine levels, can lead to long-term or irreversible functional disorders of the HPG axis. At the same time, inflammatory processes in adults induce only transient and reversible changes in HPG axis activity through the action of cytokines. In recent years, there has been a growing focus on strategies for preventing or correcting developmental disorders in the earliest stages of ontogenesis in order to safeguard offspring health.

## Figures and Tables

**Table 1:** Brief summary of cytokine effects on function and development of hypothalamic–pituitary–gonadal axis.

Cytokine	Hypothalamic Level (GnRH)	Pituitary-Level Pituitary Gonadotropins	Gonadal-Level Steroids/Gametogenesis
IL1β	Suppression of GnRH secretion in the medial preoptic area of the hypothalamus. LH surge suppression in females. Suppressive effect is mediated by an increased synthesis and secretion of GABA and a decreased norepinephrine concentration.	Suppression of LH/FSH secretion in response to GnRH.	Low doses stimulate Leydig cell proliferation High doses suppress Leydig cell steroidogenesis Reduces gonadotropin receptor expression on granulosa cells.
TNFα	Suppression of GnRH synthesis and secretion during acute and chronic inflammation in obesity or aging via KISS1 and dynorphin-expressing neurons. Suppression of KISS1 receptor expression on GnRH neurons.	Suppressive effect on LH secretion via GnRH pulse regulation.	TNFα is synthesized by testicular macrophages and spermatids and is essential for Leydig cell viability and steroidogenesis. Stimulates androgen receptor expression in Sertoli cells Suppresses anti-Müllerian hormone synthesis in them. Elevated TNFα levels suppress steroidogenesis. TNF suppresses aromatase expression in cumulus cells and adipose tissue, suppressing testosterone to estradiol conversion. TNF induces granulosa cell apoptosis and mediates ovulation.
IL-6	In adults, evidence of IL-6 effects on GnRH neurons is insufficient. In fetus, IL-6 is essential for axonal growth regulation. IL-6 elevation suppresses GnRH neuron migration into the fetal brain.	Stimulates the secretion of LH and FSH and pituitary cell proliferation.	Dose-dependent suppression of the steroidogenic function of Leydig cells, regulation of their viability. Regulates permeability of blood–testis barrier. In ovary, IL-6 mediates ovulation.
LIF	In adults, LIF stimulates GnRH secretion In fetus, LIF stimulates GnRH neuron chemotaxis.	Regulates fetal pituitary cell differentiation pathway.	LIF is essential for germ cell proliferation.
IFNγ	Evidence is insufficient. IFNγ stimulates monoaminergic activity in paraventricular nucleus, which regulates GnRH neuron activity.	TNFα suppresses pituitary hormone response to hypothalamic releasing hormones, including GnRH; Receptors for TNFα are found on endocrine cells of the anterior pituitary.	IFN induces granulosa cell apoptosis.
IL-2	Data is insufficient.	Suppression of LH and FSH secretion Stimulation of ACTH and thyreotropin secretion.	Data is insufficient.
IL-8	Data is insufficient.	IL-8 deficiency leads to LH and FSH deficiency.	Enhances Leydig cell viability and growth.
IL-10	GnRH neurons express IL-10 receptor.	Evidence of IL-10 action mechanism in estrous cyclicity maintenance is insufficient.	IL-10 deficiency is associated with estrous cyclicity suppression and disorders of fertilization and gestation.
Activin, inhibin, follistatin	Activin, inhibin and follistatin balance regulates KISS1 expression.	Activin stimulates FSH synthesis, while follistatin suppresses it.	Activin regulates the response of Sertoli cells to FSH. The balance of activin and inhibin expression by Sertoli cells depends on the stage of spermatogenic epithelium development.
Leptin	Leptin receptor is not found on GnRH neurons. Leptin regulates GnRH neurons via premamillary neurons connected with KISS1 neurons.	Leptin and other adipokine receptors are found on gonadotropocytes.	Suppression of testosterone secretion in testes. Suppression and stimulation of follicle growth depending on its concentration.

## Data Availability

No new data were created or analyzed in this study. Data sharing is not applicable to this article.

## Abbreviations

CNS	Central nervous system.
ED	Embryonic day.
ER	Estrogen receptor.
FSH	Follicle-stimulating hormone.
GABA	Gamma-aminobutyric acid.
GM-CSF	Granulocyte macrophage colony-stimulating factor.
GnRH	Gonadotropin-releasing hormone.
HGF/SF	Hepatocyte growth factor/scatter factor.
HPG	Hypothalamic–pituitary–gonadal (system).
IFNγ	Interferon gamma.
IgG	Immunoglobulin G.
IL	Interleukin.
KISS	Kisspeptin.
LH	Luteinizing hormone.
LIF	Leukemia inhibitory factor.
LPS	Lipopolysaccharide.
MCP-1	Monocyte chemoattractant protein-1.
NFkB	Nuclear Factor kappa B.
SDF-1	Stromal cell-derived factor 1 and one of its receptors CXCR4.
TGFβ	Transforming growth factor beta.
TLR	Toll-like receptors.
TNF	Tumor necrosis factor.
